# Off-Targeting of Base Editors: BE3 but not ABE induces substantial off-target single nucleotide variants

**DOI:** 10.1038/s41392-019-0044-y

**Published:** 2019-04-12

**Authors:** Huhu Xin, Tao Wan, Yuan Ping

**Affiliations:** College of Pharmaceutical Sciences, Zhejiang University, Hangzhou, 310058 China

**Keywords:** Gene therapy, Molecular medicine

Two recent articles^1,2^ published in *Science* separately reported, for the first time, that a cytosine base editor but not an adenine base editor could induce considerable off-target effects.

The advent of CRISPR-Cas9 as an RNA-guided site-specific DNA endonuclease facilitates genome modification in a precise and efficient way and brings a new dawn to targeted gene therapy.^3,4^ However, this genome editing tool has suffered from controversy due to its off-target effects. In 2016, based on CRISPR-Cas9, David Liu and coworkers developed the first cytosine base editing tool (CBE, a cytosine base editor which can readily mutate a G·C base pair to an A·T base pair), which marked a second inflection point in the advancement of genome-modifying capabilities.^5^ The following year, they established another base editing tool (ABE, adenine base editor) that can convert an A·T base pair into a G·C base pair.^6^ Compared to CRISPR-Cas9, base editors that can modify the genome without forming DSBs (double-stand breaks) are supposed to have few off-target effects. Although several methods have been developed for detecting gene-editing off-target effects through whole-genome sequencing,^7,8^ these methods are not applicable to base editors.

**Fig. 1:**
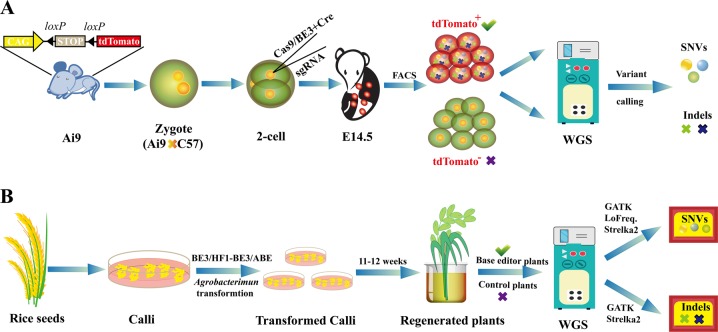
Experimental designs of Yang’s (**a**) and Gao’s (**b**) works. **a** Ai9 is a transgene mouse strain that can express robust tdTomato fluorescence following Cre-mediated recombination. A mixture of Cas9 mRNA, BE3 mRNA or ABE7.10 mRNA, sgRNA and Cre mRNA was injected into one blastomere of a 2-cell embryo. The tdTomato^+^ and tdTomato^-^ cells from E14.5 embryos were isolated by flow cytometry (FACS). Then, the two groups of cells were sequenced by WGS (whole genome sequencing) and analyzed by different algorithms. **b** Plant expression vectors of BE3, high-fidelity BE3 (HFI-BE3) or ABE were introduced into rice callus cells by *Agrobacterium*-mediated transformation, and then the base-edited plants and control plants were examined, similar to Yang’s work

Two recent papers in ***Science*** concern off-target detection for base editors. For the first time, two Chinese research groups separately reported the detection of CBE and ABE genome-wide off-target effects in mouse embryos and rice plants (Fig. 1), and they came to similar conclusions that BE3 but not ABE induces substantial off-target single nucleotide variants (SNVs).

The first study was mainly accomplished by Hui Yang’s group from Shanghai Institutes of Neuroscience, Chinese Academy of Sciences (CAS)^1^. They developed a new method named Genome-wide Off-target analysis by Two-cell embryo Injection (GOTI) to evaluate off-target mutations by editing one blastomere of a two-cell mouse embryo using either CRISPR-Cas9 or a base editor. How to eliminate nonspecific background interference is critical to obtain better results for off-target analysis throughout the genome. This work edited only one blastomere of two-cell mouse embryos, leaving the other one unedited. Meanwhile, the progeny cells of the edited blastomere were labeled with red fluorescence (tdTomato) to facilitate their separation from unedited cells. The two groups of cells possessed the same genetic background, as they were from the same zygote. By this ingenious design, nonspecific interference, such as SNPs (single nucleotide polymorphisms), can be reduced to a great extent between different samples. Using GOTI, they evaluated the off-target effects induced by CRISPR-Cas9, cytosine base editor 3 (BE3, rAPOBEC1-nCas9-UGI) and adenine base editor 7.10 (ABE7.10, TadA-TadA*-nCas9). After whole-genome deep sequencing (47 × ) (tdTomato^+^ and tdTomato^-^ cells were examined separately), they used three variant calling algorithms to analyze SNVs and indels (Mutect2, LoFreq and Strelka for SNVs, and Mutect2, Scalpel and Strelka for indels). Only the overlapping SNVs or indels identified by all three algorithms were considered true variants. Comparison of the edited *vs*. unedited cells of mouse embryos revealed that off-target SNVs were occasional in embryos edited by CRISPR-Cas9 or ABE7.10, with a frequency comparable to the rate of spontaneous mutation.

Surprisingly, on average, 283 SNVs/embryo in BE3-treated embryos were found, a level at least 20 times higher than that observed in control embryos. The authors also found that the BE3-induced off-target SNVs were sgRNA-independent, which is likely caused by the overexpression of APOBEC1. Superior to other previous off-target studies where large pools of cells were used, GOTI analyzes the cell population inherited from a single gene-edited blastomere and can avoid population averaging.

Another similar work was published in the same issue by the Caixia Gao group and coworkers from the Institute of Genetics and Development Biology, CAS.^2^ For the first time, they analyzed the off-target effects of three base editors (BE3, high-fidelity BE3 and ABE) through whole genome sequencing in rice plants. Similar to Yang’s findings, they also found that BE3 and high-fidelity BE3, but not ABE, induce considerable genome-wide off-target mutations. Notably, treatment of rice with BE3 or high-fidelity BE3 in the absence of sgRNA also leads to increased levels of genome-wide SNVs.

The above two works collectively indicate that the current cytosine base editor may induce serious off-target effects in both animals and plants, while the adenine base editor has higher fidelity. Additionally, Yang’s group found that some BE3 SNVs were located in proto-oncogenes and tumor suppressor genes, raising safety concerns about the potential oncogenic risk of BE3 for therapeutic genome editing. Therefore, BE3 needs to be optimized for high fidelity to ensure safe clinical translation for future gene therapy.
